# Asymmetrical Effects of Sleep and Emotions in Daily Life

**DOI:** 10.1007/s42761-022-00112-x

**Published:** 2022-04-07

**Authors:** David B. Newman, Elissa S. Epel, Michael Coccia, Eli Puterman, Aric A. Prather

**Affiliations:** 1 University of California, 3333 California St., San Francisco, CA 94118 USA; 2 University of British Columbia, Vancouver, Canada

**Keywords:** Sleep, Affect, Daily events, Daily diary

## Abstract

Sleep is an important process that can influence and be influenced by daily events and emotions. We examined the bidirectional relationships between sleep, daily events, and emotions with a daily diary method completed by 181 mothers (*M*_age_ = 41.91, *SD* = 5.06). They answered morning and evening questionnaires for 1 week at three different points in time separated by nine months each, 21 days in total. Measures of sleep quality and emotional experiences each morning were assessed, and they reported on their best and worst experience of the day, peak emotional responses to these events, and affect in the evening. Sleep behavior, including total sleep time and sleep efficiency, was objectively quantified using wrist actigraphy. Multilevel modeling analyses showed that longer sleep duration and better subjective quality predicted greater positive emotions and lower negative emotions upon waking, and lower levels of peak perceived stressfulness, but not peak positivity ratings. Daily experiences did not predict sleep duration. Conversely, negative affect in the evening and greater peak perceived stressfulness during the day predicted worse sleep quality that night, whereas positive affect and positive events were not related to sleep. Although correlational, these findings suggest that good sleep can improve waking affect and help mitigate the impact of stressful experiences but does not amplify responses to the positive events of the day. In turn, daily perceived stress reactivity impairs sleep quality. These novel findings show stronger bidirectional relationships between sleep with daily stress, than sleep with daily positivity.

**Supplementary Information:**

The online version contains supplementary material available at 10.1007/s42761-022-00112-x.

Sleep has important consequences on the experiences of stress and emotions as they unfold in daily life. Cross-sectional studies have shown that people who sleep more or better tend to experience more positive affect and less negative affect compared to poorer sleepers (Åkerstedt et al., 2002; Baglioni et al., 2010; Goldstein & Walker, 2014; Palmer & Alfano, 2017). Experimental studies have documented similar findings, such that a total night of sleep deprivation or partial sleep deprivation (e.g., 4 h/night) can result in increased negative emotions, such as anxiety, stress, and sadness (Kahn et al., 2013; Meerlo et al., 2008; Palmer & Alfano, 2017). Although informative, cross-sectional and experimental methods are limited in their abilities to elucidate the degree to which sleep can modulate affective experiences that occur naturally in daily life (Newman & Stone, 2019; Diener et al., 2022). Further, the links between sleep, stress, and emotion are likely bidirectional (Walker & Harvey, 2010), a process that cannot be easily or effectively tested by cross-sectional or experimental methods. To better address these concerns regarding ecological validity, researchers have turned to daily diary studies (Konjarski et al., 2018).

A growing body of research has examined the potential bidirectional relationships between sleep and affective processes. In several diary-based studies, longer sleep duration and better sleep quality have predicted greater positive affect and less negative affect on the following day (de Wild-Hartmann et al., 2013; Mccrae et al., 2008; Sin et al., 2017, 2020; Totterdell et al., 1994), although some studies have reported mixed results (Kalmbach et al., 2014).

The effect of daily affect on subsequent sleep has also yielded mixed results (Pereira et al., 2013, 2016; Winzeler et al., 2014). Some studies have found that positive affect predicts better sleep that night (e.g., Kalmbach et al., 2014). However, among insomniacs, positive affect in the evening can lead to greater sleep disturbances (Talbot et al., 2012). Other studies have shown that stress and rumination predict worse sleep (Åkerstedt et al., 2012; Garde et al., 2012; Morin et al., 2003), whereas others have documented null effects (de Wild-Hartmann et al., 2013). Some of the null effects may be attributed to the measurement of sleep and/or affect occurring after a delay (e.g., recalling yesterday’s sleep in the evening; Lee et al., 2017). See Konjarski et al. (2018) for a review.

In addition to the bidirectional associations between sleep and emotions, sleep can have important consequences on positive and negative events experienced during the day. Daily diary studies that have attempted to address these relationships have shown that subjective reports of sleep quality (Sin et al., 2017) and sleep duration (Sin et al., 2020) predict a lower likelihood of experiencing a stressor on the next day. Indeed, Lee et al. (2017) found that shorter sleep duration and poorer subjective sleep quality predicted greater reports of next-day work-to-family conflict. However, to our knowledge, no studies have examined the effect of sleep on the intensity or severity of the stressors and positive events. Nevertheless, based on prior theoretical work (Gordon et al., 2017), we hypothesized that poor sleep would lead to higher ratings of severity of negative events. Sleep may influence reactions to and perceptions of stressful events via impaired executive functioning. For example, people who sleep poorly rely heavily on automatic processing are limited in their ability to self-regulate their emotions, and may react impulsively to stressful events (Ghumman & Barnes, 2013; Krizan & Hisler, 2016; Mauss et al., 2013). Moreover, lack of sleep can hinder people’s ability to focus and pay attention to subtle social cues which could lead to more intense negative reactions in social situations (Lim & Dinges, 2010). Limited research has provided a theoretical framework for the effect of sleep on the intensity of positive events, so we examined these effects in an exploratory manner.

The goal of our study was to examine the bidirectional relationships between sleep and positive and negative emotions and to examine the effect of sleep on the levels of intensity of positive and negative events during the day. We conducted a daily diary study and sought to address several shortcomings of prior research in a few key ways. First, to limit recall bias, we measured subjective reports of sleep quality and affect shortly after they were experienced. That is, sleep quality and morning affect were assessed in questionnaires distributed in the morning, and evening affect was assessed in questionnaires distributed in the evening. Second, we used actigraphy to record objective indicators of sleep duration and efficiency. Third, to increase the generalizability of our findings beyond a single period of time, participants completed three one-week diaries separated by 9 months each. This ensures that the findings cannot be attributed solely to any abnormalities or anomalies of a few days or single week. Fourth, we captured subjective ratings of the intensity of positive and negative events of the day as well as objective ratings through coding by research assistants of the written descriptions of the positive and negative events. These methodological advantages allowed us to provide insights into the bidirectional relationships between sleep, emotions, and daily events that prior research has not been able to address as comprehensively.

## Method

Data, analytic code, and materials are available at the OSF link: https://osf.io/uqvsm. The Institutional Review Board at the University of California, San Francisco, approved the study protocol, and all participants provided written informed consent. The study was not formally preregistered.

### Participants and Procedure

Participants were 183 mothers recruited from the San Francisco Bay Area via schools, parenting publications, social media, mailings, and through child development centers as well as direct recruitment at the University of California, San Francisco Autism Clinic. A total of 183 mothers were recruited for this study; however, 181 were available for analysis because two participants did not complete an adequate number of daily reports (see Data Cleaning and Analytic Strategy below). Descriptions of this sample have been published elsewhere (Crosswell et al., 2020; Felder et al., 2018). Briefly, eligible participants were non-smokers between the ages of 20 to 50 years, with at least one child between the ages of 2 and 16 years. Roughly half of the sample were considered high-stress maternal caregivers, while the other half were low-stress maternal controls. Since caregiving was not a focus in this study, we collapsed across group status^1^. All participants reported being premenopausal and in good general health free from major diseases, including history of coronary heart disease, endocrine disorders, epilepsy, brain injury, autoimmune conditions, severe asthma, or lung disease. Structured Clinical Interviews for Diagnostic and Statistical Manual for Mental Disorders for Axis I Disorders (SCID) were carried out during the eligibility period and individuals with current psychiatric conditions, including bipolar disorder, post-traumatic stress disorder, and eating disorders were excluded. Low-stress controls who met diagnostic criterial for major depression were also excluded; this was not exclusionary for high-stress caregivers. Six of the participants (3.3%) recorded scores greater than 33 on the Inventory for Depressive Symptomatology Scale, indicating major depressive disorder (Rush et al., 2003). All participants were free from medications known to affect the immune and endocrine system with the exception of oral contraceptives. Antidepressant medication was exclusionary for low-stress controls but not high-stress caregivers. Full descriptive statistics of the participants are presented in Table 1.

**Table 1:** Participant descriptive statistics reported as M (*SD*) or % (*N*)

Variable	M (*SD*) or % (*N*)
Age	41.91 (5.06)
Race/Ethnicity	
Non-Hispanic White	76.24% (138)
Non-Hispanic Black	3.31% (6)
Non-Hispanic Asian/Pacific Islander	9.94% (18)
Non-Hispanic Native American	.55% (1)
Non-Hispanic Other/Multiracial	2.76% (5)
Hispanic or Latina	7.18% (13)
Income > $100,000	77.22% (139)
Body mass index	25.31 (4.94)
Married	87.78% (158)
Antidepressant medication use	7.18% (13)
Baseline perceived stress	18.82 (5.49)
Number of children	1.90 (.83)

As part of this study, participants completed nightly diaries for 7-consecutive nights around each of the four laboratory visits (baseline, +9 months, +18 months, +24 months follow-up). Evening questionnaires were sent by email at the preferred time for the participants, typically around 8:00 p.m. Questionnaires completed more than a day late were not included in the analyses. Morning questionnaires were completed on paper and were returned to the research team at the end of the study. Between the +18- and +24-month follow-up timepoints, a subsample of participants enrolled in a stress-reduction intervention. As such, these analyses will be constrained to data collected at the first three study timepoints. We use the term “burst” to refer to each timepoint. Thus, we analyzed data from three bursts.

### Measures

#### Sleep

Objective and subjective measures of sleep were obtained. Sleep behavior was measured using wrist actigraphy. Each participant wore an Actiwatch-2 (Philips Respironics, Bend, OR) continuously on the non-dominant wrist for 7 consecutive days at the three study time points (a total of 21 possible nights). Data were stored in 15-s epochs and scored using validated Minimitter software algorithms to estimate sleep parameters of interest (Actiware Version). Rest intervals were determined using morning sleep diaries that were collected concurrently with actigraphy assessment. Specifically, rest intervals were defined based on responses to the following questions: “What time did you try to go to sleep last night (lights out)?” and “What time did you wake up today?” Total sleep time (TST) and sleep efficiency were our sleep measures of interest. Total sleep time was defined as the total number of minutes scored as sleep by the software algorithm within the determined rest interval. Estimates of sleep continuity were assessed using the sleep efficiency variable, which was defined as the total sleep time divided by the total amount of time spent in bed. This proportion was multiplied by 100, and higher scores indicated greater sleep continuity.

Subjective sleep quality was assessed each morning as part of the morning sleep diary. Specifically, participants responded to the question, “How would you rate the quality of your sleep last night?” Responses were recorded on a 4-point scale (1 = *Very Bad*, 2 = *Fairly Bad*, 3 = *Fairly Good*, 4 = *Very Good*).

#### Affect

Positive and negative affect were assessed in the morning and evening. In the morning diaries, participants reported their levels of negative affect by responding to the statement, “I feel stressed, anxious, overwhelmed.” Positive affect was assessed with the statement, “I feel joyful, glad, happy.” Responses were recorded on a 5-point scale (1 = *Not at all*, 2 = *A little bit*, 3 = *Somewhat*, 4 = *Moderately*, 5 = *Extremely*).

In the evening diaries, participants reported their evening negative and positive affect using the modified Differential Emotions Scale (mDES; Fredrickson et al., 2003). Participants were instructed to indicate how much they felt each emotion this evening. Responses were reported on a 5-point scale (0 = *Not at all*, 1 = *A little bit*, 2 = *Somewhat*, 3 = *Moderately*, 4 = *Extremely*). Negative and positive affect composite variables were created by averaging the 12 negative and 12 positive valence items, respectively. We followed the procedure outlined by Nezlek (2017) to calculate the reliability estimates of the positive and negative affect measures. In three-level multilevel models, items were nested within days, which were nested within persons. The reliability of the intercept of the unconditional models provides an estimate of the true variance divided by total variance. The reliabilities of positive and negative affect were .85 and .70, respectively.

#### Daily Stressors and Positive Events

In the evening diaries, participants reflected on their day and provided information about their most stressful and their most positive event. Following a modified procedure by Almeida et al. (2002), participants were asked to, “Please describe, with as many details as possible, the event in your life that caused you the most stress today. We are interested in what actually happened, in other words how the event unfolded.” Participants wrote a brief description of this event. Next, they were asked, “How stressful was this situation for you, today, at its peak?” Responses were recorded on a 100-point slider scale with the labels “Not at all,” “Somewhat,” and “Extremely” at the endpoints and midpoint.

Descriptions of the reported stressful events were objectively coded for stressor severity by two independent coders (for details, see Almeida et al., 2002; Catalino et al., 2017; Crosswell et al., 2020). Events were coded on a 5-point scale (0 = *No Stressor Occurred*, 1 = *Low Severity Event*, 2 = *Medium Severity Event*, 3 = *High Severity Event*, 4 = *Extreme Severity Event*). The coders were instructed to rely on the disruption and/or unpleasantness of the events as they rated the severity. Examples of each category were provided to the coders before they began the task. The exact wording of the instructions is presented in Supplemental Materials. Coders were trained extensively prior to beginning the task such that they were required to reach 80% reliability with the team of coders before beginning their coding. Coder discrepancies were resolved by a master coder (research psychologist).

Participants were also asked to report on their most positive event of the day. Specifically, they received the following instructions: “We’d also like to know if there are situations in your life that brought you positive emotions today. Briefly, what was the most positive event or situation in your life today?” After writing a description of the event, they were asked, “How positive did this situation make you feel, today, at its peak?” Responses were recorded on a 100-point slider scale with the labels, “Not at all,” “Somewhat,” and “Extremely” at the endpoints and midpoint.

Descriptions of the positive events were objectively coded for the intensity of the positive event by two independent coders using a 4-point intensity scale (1 = *Low intensity*, 2 = *Medium intensity*, 3 = *High intensity*, 4 = *Extreme intensity*). Intensity scores were based on degrees of happiness, pleasantness, and/or restoration that the event engendered. Pleasantness was defined as the level of positive feelings or likelihood of positive outcomes generally expected to be evoked by the event. Restoration was defined as the extent to which the event had the ability to restore health, strength, or a feeling of well-being. Examples of each category were provided to the coders. Complete details of the instructions are presented in Supplemental Materials. Similar to the stressor severity ratings, coder discrepancies of the positive events were resolved by a master coder (research psychologist).

### Data Cleaning and Analytic Strategy

The assumption in daily diary methods is that the days represent a random and somewhat representative sample of days from the year (Newman & Stone, 2019). Participants who only complete a small number of daily questionnaires may provide biased responses. Therefore, we eliminated data from a particular burst from 10 participants who provided less than three daily reports during the 1-week burst period. Of the initial sample of 183 participants, 2 did not complete at least three valid entries during any of the three bursts, 22 completed one valid burst, 16 completed two valid bursts, and 143 completed all three valid bursts. Participants completed an average of 6.69 (*SD* = .73) valid evening questionnaires and an average of 6.83 (*SD* = 1.08) morning questionnaires during a typical week. Overall, compliance was very good and similar to many daily diary studies (see Nezlek, 2012, for a discussion).

Given the nested structure of the data, we used multilevel modeling to account for the different types of variances. We conceptualized the data as a three-level structure, with days nested within bursts, and bursts nested within persons.^2^ We used the *lme4* package in R (Bates et al., 2015) and trimmed random effects if the variances were particularly low and if the model failed to converge (see Nezlek, 2012, for a discussion of similar practices).

At a descriptive level, we created null or unconditional models which provide estimates of the means and variances at each level for each of the daily measures. Next, we ran four sets of models (resulting in 36 models in total) to test the primary research questions. The first two sets of models examined the consequences of sleep, and the final two sets of models examined the antecedents of sleep. The first question addressed the effect of sleep on positive and negative affect in the morning. The second question addressed the effect of sleep on ratings of the most stressful and most positive events of the day. The third question addressed the effect of positive and negative affect experienced in the evening on sleep that evening. The fourth question addressed the effect of stressor severity and positive event intensity on sleep that evening. In each model, the predictor variables were centered around the average score for that participant during that particular burst. This removes between-person and between-burst variation to provide a pure estimate of the within-person relationships, as recommended by Enders and Tofighi (2007).

## Results

### Descriptive Statistics of Daily Measures

The results from unconditional models in which there are no predictors at any level showed that most of the variation for each variable occurred within-persons within-burst (see Table 2). This means that an individual’s sleep, morning and evening affect, and the intensity/severity of positive events and daily stressors varied quite a bit from one day to the next. This reiterates the importance of examining within-person daily relationships. Some variation occurred between-persons, indicating that some individuals sleep better than others and experience events and emotions differently from others on average. Very little variation occurred within-persons between-bursts, which indicates that average levels of sleep, events, and affect remained relatively stable for each person over the 18 month-period.

**Table 2:** Descriptive statistics of daily measures

Variable	Mean	Variation			ICCs	
			Within-person			
		Between-person	Between-burst	Within-burst	Person-level ICC	Burst-level ICC
Sleep						
Total sleep time (minutes)	405.81	1230.20	313.50	4134.20	.22	.27
Sleep efficiency (%)	87.87	5.52	13.89	46.99	.08	.29
Sleep quality (1–4 scale)	2.85	.11	.03	.45	.19	.24
Morning affect						
Morning positive affect (1–5 scale)	2.96	.50	.11	.53	.44	.54
Morning negative affect (1–5 scale)	2.07	.34	.11	.53	.35	.46
Event ratings						
Peak positivity of positive event (0–100 scale)	79.77	80.13	6.76	137.68	.36	.39
Intensity of positive event (RA coded) (1–4 scale)	1.88	.07	.02	.37	.15	.20
Peak stressfulness of negative event (0–100 scale)	56.51	178.46	24.46	427.30	.28	.32
Severity of negative event (RA coded) (0–4 scale)	1.53	.06	.04	.41	.12	.20
Evening affect						
Evening positive affect (0–4 scale)	1.93	.30	.07	.34	.42	.52
Evening negative affect (0–4 scale)	.50	.10	.02	.14	.38	.46

### Sleep Predicts Morning Affect

To examine the effect of sleep on morning affect, we entered total sleep time, sleep efficiency, and sleep quality as predictors at level 1 in separate models. Positive and negative affect were outcome measures in separate models as follows:

Day level: *y*_*ijk*_ (morning affect) = π_0*jk*_ + π_1*jk*_ (sleep) + *e*_*ijk*_

Burst level*:* $$ {\displaystyle \begin{array}{c}{\uppi}_{0 jk}={\upbeta}_{00k}+{r}_{0 jk}\\ {}{\uppi}_{1 jk}={\upbeta}_{10k}+{r}_{1 jk}\end{array}} $$

Person level: $$ {\displaystyle \begin{array}{c}{\upbeta}_{00\mathrm{k}}={\upgamma}_{000}+{u}_{00k}\\ {}{\upbeta}_{10\mathrm{k}}={\upgamma}_{100}+{u}_{10k}\end{array}} $$

As shown in the top portion of Table 3, longer total sleep time predicted greater positive affect and lower negative affect in the morning. Effect size estimates that are presented alongside these coefficients indicated that the effect sizes were small to medium in magnitude. Similarly, better sleep quality relative to one’s typical sleep quality predicted greater morning positive affect and lower morning negative affect. Sleep efficiency was not significantly related to morning positive affect but significantly predicted lower morning negative affect. In sum, as someone sleeps longer or better than they typically do, they report higher levels of positive affect and lower levels of negative affect in the morning. Additionally, better sleep continuity relative to an individual’s typical level was associated with lower morning negative affect.

**Table 3:** Sleep predicts morning affect and daily event ratings

	Predictors											
Outcome measures	Total sleep time				Sleep efficiency				Sleep quality			
Morning affect	*b*	*t*	*p*	*r*	*b*	*t*	*p*	*r*	*b*	*t*	*p*	*r*
Positive affect	.001	5.34	< .001	.18	.002	.86	.390	.12	.20	8.63	< .001	.21
Negative affect	−.001	−5.44	< .001	.22	−.007	−3.31	< .001	.17	−.22	−9.29	< .001	.21
Daily events	*b*	*t*	*p*	*r*	*b*	*t*	*p*	*r*	*b*	*t*	*p*	*r*
Peak positivity of positive event	.005	1.13	.258	.02	−.06	−1.40	.167	.10	.005	.01	.990	.04
Intensity of positive event (RA coded)	−.000	−.05	.962	.05	−.003	−1.48	.140	.02	.02	.84	.404	.02
Peak stressfulness of negative event	−.02	−2.85	.005	.17	−.015	−.20	.840	.09	−1.36	−2.19	.031	.03
Severity of negative event (RA coded)	−.001	−2.75	.006	.07	−.001	−.45	.657	.04	−.02	−1.21	.227	.02

Effect size estimates were calculated using a method explained by Rights and Sterba (2019). The *r* statistic is analogous to the square root of the reduction in variance method initially described by Raudenbush and Bryk (2002), akin to a correlation. Note that these effect size estimates may not correlate intuitively with *p*-values as they combine the variance associated with fixed and random effects. Separate effect sizes for each type of variance are reported in the Supplemental Materials.

These effects were not significantly moderated by group status (i.e., high stress mothers vs. low stress mothers; all *p*s > .080, four *p*s > .55).

### Sleep Predicts Daily Positive and Negative Events

In the next set of analyses, we examined whether sleep predicted the objectively coded and subjectively reported intensity/severity of the most positive and most stressful events of the day. Similar to before, each of the sleep variables were predictors at level 1, centered around each individual’s mean within each burst. The subjective ratings of the positive and negative events and the objective ratings of intensity/severity by the research assistants were outcome variables in separate models as follows:

Day level: *y*_*ijk*_ (event ratings) = π_0*jk*_ + π_1*jk*_ (sleep) + *e*_*ijk*_

Burst level*:* $$ {\uppi}_{0 jk}={\upbeta}_{00k}+{r}_{0 jk} $$

$$ {\pi}_{1 jk}={\upbeta}_{10k}+{r}_{1 jk} $$

Person level: $$ {\upbeta}_{00\mathrm{k}}={\upgamma}_{000}+{u}_{00k} $$

$$ {\upbeta}_{10k}={\upgamma}_{100}+{u}_{10k} $$

The results from these models presented in the bottom portion of Table 3 showed that total sleep time, sleep efficiency, and sleep quality were not significantly related to the ratings of peak positivity or objectively coded intensity of the most positive event of the day. In contrast, longer total sleep time predicted lower subjective ratings of peak stress to the most stressful event of the day and lower objectively coded ratings of stress intensity. That is, when participants slept longer than they typically do, the daily stressors they reported were judged by coders to be less severe and participant’s subjective perceptions of those events were attenuated. Better sleep quality predicted lower subjective ratings of peak stress in response to the daily stressor but was not significantly related to objectively coded ratings of stress severity. Sleep efficiency was not significantly related to subjective or objectively coded measures of stress. Further, these effects were not significantly moderated by group status (all *p*s > .051, most *p*s > .36). These findings suggest that a longer night of sleep or one perceived of higher quality than is typical was associated with reports of less severe, objectively coded stressful events that were perceived as less stressful by the participant than would otherwise be the case. In contrast, better, longer sleep did not relate to the intensity or subjective feelings of positivity to the most positive event of the day.

### Evening Affect Predicts Sleep

In the final two sets of models, we considered the antecedents to sleep. We first examined the influence of positive and negative affect experienced during the evening on that night’s sleep. In these models, positive and negative affect were entered as predictors in separate models^3^ and were centered around each individual’s mean within each burst. Total sleep time, sleep efficiency, and sleep quality were outcome measures in separate models as follows:

Day level: *y*_*ijk*_ (sleep) = π_0*jk*_ + π_1*jk*_ (evening affect) + *e*_*ijk*_

Burst level*: *$$ {\uppi}_{0 jk}={\upbeta}_{00k}+{r}_{0 jk} $$

$$ {\uppi}_{1 jk}={\upbeta}_{10k}+{r}_{1 jk} $$

Person level: $$ {\upbeta}_{00\mathrm{k}}={\upgamma}_{000}+{u}_{00k} $$

$$ {\upbeta}_{10\mathrm{k}}={\upgamma}_{100}+{u}_{10k} $$

As presented in Table 4, positive affect in the evening did not predict the total sleep time, sleep efficiency, or sleep quality that night. In contrast, negative affect in the evening predicted lower self-reported sleep quality but did not predict total sleep time or sleep efficiency. Thus, positive affect in the evening did not predict better or more sleep, whereas negative affect in the evening predicted lower reports of sleep quality the following morning. These effects were not significantly moderated by group status (all *p*s > .081; most *p*s > .37).

**Table 4:** Evening affect and daily events predict sleep on that evening

	Predictors							
	Evening positive affect				Evening negative affect			
Outcome measures	*b*	*t*	*p*	*r*	*b*	*t*	*p*	*r*
Total sleep time	.60	.25	.804	.11	−1.84	−.52	.607	.04
Sleep efficiency	.31	1.29	.199	.06	−.24	−.58	.562	.11
Sleep quality	.02	.86	.388	.10	−.14	−3.53	.001	.13
	Peak Positivity of Positive Event				Intensity of Positive Event (RA coded)			
Outcome measures	*b*	*t*	*p*	*r*	*b*	*t*	*p*	*r*
Total sleep time	.08	.77	.440	.10	.64	.29	.771	.04
Sleep efficiency	.00	.26	.792	.05	.05	.22	.828	.11
Sleep quality	.00	.39	.697	.02	.03	1.23	.218	.05
	Peak Stressfulness of Negative Event				Severity of Negative Event (RA coded)			
Outcome Measures	*b*	*t*	*p*	*r*	*b*	*t*	*p*	*r*
Total sleep time	.01	.23	.817	.08	3.86	1.85	.065	.04
Sleep efficiency	.01	.92	.360	.03	.40	1.58	.118	.09
Sleep quality	−.00	−2.14	.033	.11	.00	.21	.837	.12

### Daily Positive and Negative Events Predict Sleep

Finally, we examined the effects of objectively coded intensity of daily positive events and the severity of daily stressors, and subjective reports of positive affect and peak stress to each respective daily event on each sleep measure. The models were identical to the previous models with the exception that event ratings replaced evening affect scores.

Day level: *y*_*ijk*_ (sleep) = π_0*jk*_ + π_1*jk*_ (event ratings) + *e*_*ijk*_

Burst level: $$ {\uppi}_{0 jk}={\upbeta}_{00k}+{r}_{0 jk} $$

$$ {\uppi}_{1 jk}={\upbeta}_{10k}+{r}_{1 jk} $$

Person level: $$ {\upbeta}_{00\mathrm{k}}={\upgamma}_{000}+{u}_{00k} $$

$$ {\upbeta}_{10\mathrm{k}}={\upgamma}_{100}+{u}_{10k} $$

These models showed that subjective peak stress to the most stressful event of the day predicted poorer sleep quality that night but was not associated with total sleep time or sleep efficiency. All other effects were not statistically significant (see Table 4). These effects were not moderated by group status (all *p*s > .15) with one exception^4^.

## Discussion

Using a daily diary method completed by women from ages 20 to 50 across a range of stress levels, we evaluated the bidirectional relationships between sleep, emotions, and daily events. Our results revealed several interesting asymmetrical^5^ patterns with one exception. Total sleep time and sleep quality had roughly equal effects on morning affect: Each predicted greater positive affect and lower negative affect upon waking.

We also tested the relationship between sleep and affective reactivity to daily events. In an asymmetrical pattern, total sleep time and sleep quality predicted lower levels of perceived stressfulness of the most stressful event of the day, but they were not related to the perceived positivity of the most positive event of the day. Thus, insufficient sleep appears to intensify the negative aspects of the day, while one’s experience of positive events appear resilient to poor sleep. Although precise causal mechanisms could not be tested, our results dovetailed with potential mechanisms proposed by prior research (Kahn et al., 2013). For example, sleep deprivation may hinder successful emotion regulation strategies (Payne et al., 2012), and REM sleep may help ameliorate negative emotional experiences (Levin & Nielsen, 2009; Walker, 2009; Walker & van der Helm, 2009).

We also observed asymmetrical effects when it came to the influence of stress and affect on that night’s sleep. Negative affect in the evening predicted worse sleep quality that night, but positive affect was not related to sleep. Similarly, the perceived stressfulness of the most stressful event of the day predicted worse sleep quality that night, but the positivity of the most positive event of the day was not related to sleep that night. Thus, our results are consistent with the notion that the negative aspects of the day impair subjective ratings of sleep quality, whereas the positive aspects of the day do not appear to influence sleep. One reason for this pattern of effects is that negative affect in the evening may elevate somatic and cognitive arousal which can interfere with the ability to sleep (Winzeler et al., 2014; see also Gordon et al., 2017).

To integrate the findings, it can be helpful to situate them within the context of two pertinent theories/principles: conservation of resources (COR) theory (Hobfoll, 1989) and the “bad is stronger than the good” principle (Baumeister et al., 2001). COR posits that people have a natural tendency to accumulate resources that can be used to overcome or respond to stress and threats (Hobfoll, 1989). If resources (e.g., sleep) are lacking, people are less able to effectively handle stressors and negative situations (Barnes et al., 2012; ten Brummelhuis & Bakker, 2012). Without adequate sleep, people may not be equipped with the resources necessary to mitigate the negative effects of daily stressors. The effects of sleep on the perceived stressfulness of the most stressful event of the day are consistent with a COR framework. That is, sleep provides resources that people can use to combat the negative experiences of the day.

According to the principle that the bad is stronger than the good, negative events, experiences, and emotions have a stronger influence on outcomes than positive ones (Baumeister et al., 2001). Support for the asymmetrical effects of positive and negative experiences has been documented across a range of areas in psychological research, including daily well-being (David et al., 1997; Nezlek & Gable, 2001; Sheldon et al., 1996). This theory predicts that sleep should be more heavily influenced by negative experiences than positive ones. Our results were consistent with this prediction. Stressors during the day and negative affect in the evening predicted lower subjective sleep quality, but positive events and positive affect in the evening were not related to sleep quality. Consistent with an evolutionary perspective, negative events and experiences signal that something needs our attention, and this could interfere with the brain’s ability to sleep well. Overall, COR serves as a useful theory in determining the effects of sleep on daily emotions and events, whereas the bad is stronger than the good principle can be helpful in explaining the effects of daily events and emotions on sleep.

We found that most of the relationships involving daily events and emotions were most robust with total sleep time and sleep quality, with fewer significant associations observed with sleep efficiency. Sleep quality was measured subjectively as were the emotions and daily events; hence, the processes that influenced the self-report judgments of sleep quality may have been similar to the processes that influenced the self-report judgments of emotions and daily events (Schwarz & Strack, 1999). Interestingly, total sleep time was assessed behaviorally using wrist actigraphy and had similar relationships with morning affect and daily events as did subjective sleep quality. In contrast, sleep efficiency, which was also assessed objectively with actigraphy, was often not significantly related to emotions or daily events. The links between total sleep time and our study outcomes are consistent with experimental studies showing that acute sleep loss can amplify negative emotion in response to stress. Further, this study is in line with others that emphasize the importance of measuring distinct aspects of sleep as they provide unique information (Gordon et al., 2017, 2019; Jackowska et al., 2016; Jarrin et al., 2013).

Most of the relationships between sleep and affect were consistent with prior research. Most prior daily diary and EMA studies have shown that various indicators of sleep predict greater levels of positive affect and lower levels of negative affect in the morning (Bouwmans et al., 2017; Garde et al., 2012; Mccrae et al., 2008; Sin et al., 2017, 2020). Conversely, the effects of daytime affect on sleep in the evening have yielded more mixed effects. Similar to our findings, some studies have reported stress in the evening predicting worse sleep quality (Åkerstedt et al., 2012; Garde et al., 2012; Morin et al., 2003), whereas other studies have documented null or mixed effects (Bouwmans et al., 2017; de Wild-Hartmann et al., 2013; Sin et al., 2017; Winzeler et al., 2014). Reasons for some of the discrepant findings could be due to the measurement of affect and sleep at times that required lengthier recalls (e.g., reflecting on prior night’s sleep quality in the evening) or the use of solely subjective reports. Our study may have detected these effects with greater sensitivity given that sleep quality was measured immediately upon waking, over a longer period.

To our knowledge, this is the first study to examine the relationships between sleep and ratings of intensity/severity of positive and negative events by independent coders. Studies that have measured the frequency of positive and negative events have documented results similar to ours, namely, that better sleep predicted fewer stressors on the following day (Sin et al., 2017, 2020). Analyses examining the effect of sleep on the frequency of positive events have yielded mixed (or at least not as consistent) findings. This discrepancy may be due to differences between the frequency and intensity of the positive events.

### Limitations

The study sample was limited to mothers with young children, which limits generalizability. There is growing evidence of sex differences in sleep (Becker et al., 2018; Mong & Cusmano, 2016), and it is possible that insufficient sleep may have a stronger influence on stress and emotions in one sex than the other. The sample was limited in age (range: 20–50). Future research could fruitfully include a wider range of ages as sleep may influence emotions and daily events quite differently in teenagers or children or as people get older (Hoch et al., 1997; Monk et al., 1994). For example, older adults may be less affected by poor sleep than younger adults (Philip et al., 2004; Schwarz et al., 2019) and hence may react less strongly to negative stressors during the day. In addition, participants were, in general, highly educated and fairly affluent. As such, the types of emotions and daily events that people experience differ considerably across different levels of socioeconomic status (Grzywacz et al., 2004; Prather et al., 2017; Surachman et al., 2019), which could have constrained this sample. Additionally, we used single item measures of positive and negative affect in the morning diaries which limited our ability to distinguish activated from deactivated states. Finally, future research could benefit by including a range of daily positive and negative events in addition to the most stressful or most positive ones to examine how sleep influences minor daily events. These studies could also utilize Ecological Momentary Assessment designs by asking people to report on their daily events immediately as they occur to minimize any end-of-day recall bias.

## Conclusion

In summary, we examined the interrelationships between sleep, emotions, and daily events using a daily diary method with a sample of healthy mothers. Sleep mitigated the negative effects of stressful events of the day but did not predict the emotional responses or reported magnitude of positive events of the day. Conversely, negative affect in the evening and the perceived stressfulness of the most stressful event of the day predicted lower sleep quality that evening, whereas the positive aspects of the day did not predict sleep. Thus, sleep may influence and be influenced by positive and negative aspects of the day in asymmetrical patterns.

## Supplementary Information

ESM 1 (DOCX 58 kb)

## Footnotes

1 We first examined interactions across groups. Of the 36 total analyses, only one was significantly moderated by group status. This may be due to chance, and given that our focus did not center on group differences, we collapsed across conditions. We briefly note the interaction effects in the results.

2 Because there were only three bursts, it could be argued that the burst level should be ignored in favor of a more parsimonious, two-level model in which days are nested within persons. We believe, however, that the burst level provides an important structure to the data. Nevertheless, we ran all models as two-level models and report those results in the Supplemental Materials. The substantive conclusions remained the same for nearly every model.

3 We ran additional models in which evening positive and negative affect predicted sleep to account for the covariation between positive and negative affect. These results were very similar to the models with each affect measure entered separately. Furthermore, as evening affect was measured with multiple items, we were able to distinguish activated from deactivated states. These results yielded no differences between activated and deactivated states. Results from both sets of models are presented in Supplemental Materials.

4 This was the only significant moderation of the 36 total analyses presented in this paper, so it may be due to chance. Moreover, given that our focus did not center on group differences, we do not emphasize any potential theoretical significance of this finding.

5 The asymmetrical patterns we describe refer to the comparison between positive and negative affect or between positive event ratings and negative events ratings (as opposed to morning vs. evening effects).

## Additional Information

### Availability of Data and Material/Code Availability

Data, syntax, and materials are available at https://osf.io/uqvsm

### Open Practices

The data, analyses, and materials are available at the OSF (osf.io/uqvsm) for the purpose of verification of the results presented in this manuscript. The analyses were not formally preregistered.
