# Correction to: Asymmetrical Effects of Sleep and Emotions in Daily Life

**DOI:** 10.1007/s42761-022-00120-x

**Published:** 2022-05-04

**Authors:** David B. Newman, Elissa S. Epel, Michael Coccia, Eli Puterman, Aric A. Prather

**Affiliations:** 1 University of California, 3333 California St, San Francisco, CA 94118 USA; 2 University of British Columbia, Vancouver, Canada

**Correction to: Affective Science**

10.1007/s42761-022-00112-x

The publishing team at Affective Science made errors in the manuscript “*Asymmetrical Effects of Sleep and Emotions in Daily Life*.” Specifically, they did not copy some of the equations correctly and failed to convert some of the Greek symbols.

This erratum is presented to fix these errors.

The original article has been corrected.

