# Supplementary material for: Asymmetrical Effects of Sleep and Emotions in Daily Life
Source: Affect Sci. 2022 Apr 7;3(2):307–17. doi: 10.1007/s42761-022-00112-x (PMC9383029; doi:10.1007/s42761-022-00112-x)
Supplement: Supplementary file 1 — (DOCX 58 kb) [file 42761_2022_112_MOESM1_ESM.docx]

**Supplemental Materials**

**Overview**

First, we present additional descriptions of the instructions the coders received as they rated the positive and negative events. Next, in addition to the three-level models presented in the manuscript, we present the results from two-level models (days nested within persons) as well. The substantive conclusions remained the same. These results are presented in Supplemental Tables 1 – 3. In Supplemental Tables 4 – 5, we include confidence intervals, standard errors, and all effect size estimates from the three-level models presented in the main text. Next, we examined the relationships between evening affect and sleep by including positive affect and negative affect in the same model. (The analyses reported in the main text included them as predictors in separate models). See Supplemental Table 6. Finally, in addition to differentiating valence (positive affect vs. negative affect), we distinguished activated and deactivated states of evening affect using a circumplex model. These results are presented in Supplemental Table 7.

**Stressor Coding Instructions**

**SEVERITY**

**0** = no stressor occurred

**1** = low severity event

**2** = medium severity event

**3** = high severity event

**4** = extreme severity

The Four Point Severity Scheme (1, 2, 3, 4) is a measure of the degree of **disruption AND/OR unpleasantness** represented by the occurrence of an event.

The key to the distinction relies on objective evidence from the Respondent's report of the situation.

**Disruptiveness** refers to the extent to which there is a change or likelihood of a change in some aspect of an individual’s life. Disruptiveness is also the extent to which an event can affect daily routines, important plans, concerns and goals.

The **unpleasantness** of an event is the level of negative feelings, or the risk of negative consequences generally expected to be evoked by the event.

Low unpleasantness (Severity ‘1’)—Respondent is cut off by traffic and finds it difficult to merge onto the Interstate.

Medium unpleasantness (Severity ‘2’)—Respondent is caught in a hour-long traffic jam on the way to work and nearly doesn’t make it to work on time.

High unpleasantness (Severity ‘3’)—Respondent forgets to put gas in the car and runs out of gas during the traffic jam on highway; she is late to work, but her boss is understanding and the incident does not threaten her job.

Severe unpleasantness (Severity ‘4’)—Respondent arrives at work late, and the boss yells, “this is the last time you’re late, next time you’re fired! Report to personnel!”

**Severity Rating “0” – No Stress Reported**

Participant reports **no** stressor occurred that day. A participant might write “nothing bad happened”.

If the participant typed nothing in the text box, please assign the code of “-88”, which indicated missing data.

**Severity Rating “1” – Low Severity Events**

1. Low severity events are trivial occurrences or daily hassles causing slight disruption or anticipation of slight disruption to an aspect of the Respondent's life.

**OR**

1. Low severity events are occurrences that would evoke only fleeting (lasts less than an hour) unpleasant emotions or reactions for the reasonable person

Examples:

a. Everyday worry, future-thinking (e.g., worry about a schedule change or whether “something will work out” later in the day).

b. Everyday type of small events or occurrences that are annoying, frustrating, or not life-changing, such as disruptions in daily routine (e.g., problems with public transportation).

c. I had a bad run in with a compost bucket that hadn't been taken out to the bin for a while--------- SUPER GROSS!!

d. Dog almost getting away from us on a walk

e. Getting my youngest daughter to clean up her things and pack for camp

f. (from manual) “We were on the way to the jazz concert and…it was something little but it was irritating to me. I avoided arguing. She’s never on time when I come to pick her up. I drive all the way over to pick her up and I have to wait. She’s never on time. I felt good today and I wasn’t going to let her ruin my day.”

**Severity Rating “2” – Medium Severity Events**

1. Medium severity events are more disruptive than daily hassles. Medium severity events carry no potential or only a slight potential for major disruption in the future.

**OR**

1. Medium severity events are unpleasant enough to evoke unpleasant emotions for more than an hour or two for the reasonable person. Alternatively, medium severity events could make someone feel “a little bad” for the whole day.

Examples:

a. Loss, threat, or damage inflicted on the person or someone close to the person that has a negative effect on him/her, but is not life-changing (e.g., conflict with a friend).

b. More than a usual daily tantrum or a “bigger episode” that affects a larger part of their day. “My son with Autism was sad, aggressive and tantrum today after his day care program. I had already had an overwhelming day at work where I had too much to do in too little time. I didn't have much patience with him and when he pinched me I pinched him back.”

c. No news regarding my son’s soccer tryouts..some of the team have already been informed, so the probability is getting lower that he was selected. This is extremely important to him, so we are concerned about the potential disappointment for him.

d. Trying to coordinate multiple grandparents in town for a birthday party to manage their various needs and personalities.

e. My son got a bad ear ache in the late afternoon and was unhappy and in pain.

f. (from manual) “This afternoon my two youngest daughters competed in a state piano competition and that’s really stressful. I think most people would consider that stressful because it’s very tense and all the kids are pacing up and down the hall and the judges are there being stern and you’re talking to the judge and you’re trying to keep the kids making sure they do their best and everybody’s all stressed out and people snap at each other.”

**Severity Rating “3” – High Severity Events**

1. High severity events are very disruptive to the given day (e.g. an event derailing a person’s day) and could imply *a potential for future major disruption*, although this is not required. High severity events do not carry explicit threat of major disruption at the time they occur.

**OR**

1. High severity events may be "unpleasant" events, which are likely to provoke negative feelings lasting *most of the day* for the reasonable person.

Examples:

a. My aspergers high schooler could not be found at school for several periods of the day. He is a bit agitated about several new classes and resistant to school.

b. Daughter had a bullying incident at school

c. I had to call 911 today while we were meeting friends at the playground. My younger daughter and her friend couldn't be found; they were not where we thought they were playing and after looking and calling for them for 30 minutes, we found someone who said she saw them and that they had gone with a man. At that point, what had been relatively mild worry turned to full blown anxiety and I called the police. They came promptly and right at that time the two girls were found. They had been playing in another area of the park where we couldn't see them and it wasn't the area that we thought they meant when they were telling us their planned location. It was all fine, but I don't think I've ever had that level of anxiety/fear/worry in my life.

d. (from manual) “I haven’t seen my daughter for two days, she’s been over a friend’s house. Today she got home and I spent some time with her and she wanted to spend the night with somebody else. I’m actually paying this other lady to take care of my children. I feel like I’m not the mom I used to be. It really bothers me because of the situation we’re in right now”. (R’s daughter is 8 years old).

**Severity Rating “4” – Extreme severity events**

1. Extreme severity events either *immediately result in a major disruption* or carry a *high likelihood* of a major disruption occurring.

**OR**

1. Extreme severity events would be expected to generate unpleasant emotions lasting *more than a day* for the reasonable person.

Examples:

a. Potentially a life-changing event

b. A significant loss, threat, or damage to the person or someone close to the person that is in some way life changing (e.g., divorce, bereavement, going to jail, learning about spouse adultery).

c. My son’s EEG was terrible, signally recurrence of syndrome that causes regression developmentally and uncontrolled seizures (although I discovered coders coded this as a “3”—this should be changed)

d. My dad just died and I've been fighting with my spouse and not receiving the support i need. I am avoiding him.

e. (from manual) “I had a friend killed in a car accident last night. I go to school with his mother and I’ve known him since he was a small boy and he’s just 29 so, you know, it was somewhat. Maybe the shock yet. You worry about yourself because you’re on the roads too. I’m not in such a rush. Because, you know, that was a problem. He was in a rush to get to work. It’s make me more conscious. After a while, you’ll forget about it and become more careless yourself.”

f. (from manual) “Worrying about this re-organizing and some people may lose their jobs. Our warehouse is getting rid of some of our items so they won’t need to be shipped and it is causing some people to lose their jobs. We don’t know who will be the ones or when it will happen. (R doesn’t think she will be one but) “it is possible” (She doesn’t know how it will affect her income. Said maybe a little since she carries insurance for her family)

**Positive Event Coding Instructions**

**The Intensity Scheme measures the degree of happiness, pleasantness, and/or restoration engendered by the event.**

1 = Low Intensity Positive Event

2 = Medium Intensity Positive Event

3 = High Intensity Positive Event

4 = Extreme Intensity Positive Event

The **pleasantness** of an event is the level of positive feelings, or the likelihood of positive outcomes generally expected to be evoked by the event or occur as a result of the event. Examples:

- Low pleasantness: going for a walk
- Medium pleasantness: went to a meal with a friend
- High pleasantness: taking your best friend out for a birthday dinner
- Extreme pleasantness: recommended for a prestigious professional opportunity

**Restoration** refers to the extent to which the event had the ability to restore health, strength, or a feeling of well-being to the Respondent. Restoration is also the extent to which an event can restore positive feelings in daily routines, important plans, concerns, and goals.

**Low Intensity Positive Event (Intensity = 1)**

Low intensity positive events are trivial positive occurrences or typical/routine daily life positive situations causing slight restoration or anticipation of slight restoration to an aspect of the Respondent’s life. These are not expected to have a significant impact on the Respondent’s life or emotional state.

OR

Low intensity positive events are occurrences that would evoke only fleeting (lasts less than an hour) positive emotions or reactions for a reasonable person.

Examples:

1. Went for a walk
2. I was able to sleep late and have nothing scheduled today until late afternoon.
3. Playing at rehearsal
4. Session with client
5. Putting my daughter to bed
6. I got to practice flute.
7. Attending book club

**Medium Intensity Positive Event (Intensity = 2)**

Medium intensity positive events are *more* happiness and restoration than routine daily pleasant situations. Medium intensity events carry no potential or only a slight potential for major restoration in the future.

OR

Medium intensity events are pleasant enough to evoke positive/pleasant emotions for more than an hour or two for a reasonable person. Alternatively, medium intensity events could make someone feel “a little happy” for the whole day.

Examples:

1. My son asked me to play tickles this evening.
2. I went out to lunch with a friend.
3. Taking my ASD child to open his first savings account.
4. Tonight as we were driving, my son nonverbally asked me to play tickle with him. He laughed and snorted as he loved the game and attention. I love hearing him laugh and knowing he is happy.
5. getting a lot done on my to do list
6. My son made some progress with his therapist
7. worked most of the day productively, felt good to not just be there taking up space. still need to produce so while i got some data in the system, need more to show.

**High Intensity Positive Event (Intensity = 3)**

High intensity positive events are very restorative for that person on that given day (e.g., an event that improved a person’s whole day) and could imply a potential for future major positive outcomes, restoration, and happiness, although this is not required. High intensity events may not carry the explicit possibility of major restoration at the time they occur.

OR

High intensity events may be “pleasant” events, which are likely to provoke positive feelings *lasting most of the day* for a reasonable person.

Examples:

1. Someone I have been working with said she could share a grant she got with my daughters school so I was able to tell a couple of kids that they could take a gardening class I organize for free. They were very happy!
2. Our offer on a house went in after a lot of back and forth, inspection, etc
3. went to block party with family in the afternoon, saw lots of friends- fun and relaxing.
4. Went for an amazing hike in Marin with daughter and husband. Great weather. Smell of laurel in the air. No meltdowns. Husband was super affectionate. Just perfect
5. I was playing with my daughter, and she wanted to 'pretend sleep', so we snuggled up under a blanket and she closed her eyes and she had these rosy cheeks and long eyelashes, and my heart was filled with so much joy.
6. I took my best friend out for her birthday.
7. Putting my daughter to bed. She took two shells out of a box, and said she was going to do a magic spell so that we would always be together. She blew on them, rubbed them with her nose, and held them up to the stars. She said they had powerful good magic for us both, and meant that we would always be together. It was very sweet, quiet, and calm. We have been feeling disconnected this past week, so I was really glad to have quiet sweet time.

**Extreme Intensity Positive Event (Intensity = 4)**

Extreme intensity positive events either immediately result in major restoration or carry a high likelihood of positive outcomes or major restoration occurring.

OR

Extreme intensity positive events would be expected to generate positive emotions lasting more than a day for a reasonable person.

Examples:

1. A friend recommended me for a prestigious professional opportunity.
2. I was awarded a certificate of achievement at my kids school for volunteering over 400 hrs with 2 other people. It felt good to be recognized.
3. I mailed in close-to-final paperwork to refinance my house, which will put me in a much better position financially. I've been working on this for over a year, and it hasn't been clear until very recently that it would work out. It could mean a big change in my life, gives me a lot more ease and comfort about financial decisions. I feel excited about the possibilities, but nervous about making any big changes. I have been so afraid of my mortgage for so long. It is hard to believe this is happening. I feel like a big weight is lifting off my shoulders, but I still have to keep checking to make sure it is not there. I still have a vague fear something could go wrong at the last minute.
4. My husband and I went to a fancy birthday party. We danced, laughed and socialized. It was a great evening!
5. Our autism site (www.thinkingautism.com) was voted best blog at Babble.com despite fierce competition.
6. I attended a lecture at the zen center today that was very inspiring. It made me feel very good about some changes I have made in my life and helped me think holistically about some elements of my life that I had worried were out of line.

Supplemental Table 1. Descriptive Statistics of Daily Measures

|  |  | **Variation** | |
| --- | --- | --- | --- |
| **Variable** | **Mean** | **Between-person** | **Within-person** |
| Sleep |  |  |  |
| Total Sleep Time | 405.86 | 1336 | 4341 |
| Sleep Efficiency | 87.86 | 10.60 | 55.82 |
| Sleep Quality | 2.85 | .12 | .46 |
|  |  |  |  |
| Morning Affect |  |  |  |
| Morning Positive Affect | 2.95 | .54 | .61 |
| Morning Negative Affect | 2.08 | .38 | .61 |
|  |  |  |  |
| Event Ratings |  |  |  |
| Peak Positivity of Positive Event | 79.76 | 82.77 | 142.15 |
| Intensity of Positive Event (RA coded) | 1.88 | .08 | .38 |
| Peak Stressfulness of Negative Event | 56.51 | 186.60 | 443.60 |
| Intensity of Negative Event (RA coded) | 1.53 | .08 | .44 |
|  |  |  |  |
| Evening Affect |  |  |  |
| Evening Positive Affect | 1.93 | .33 | .39 |
| Evening Negative Affect | .50 | .11 | .15 |

Supplemental Table 2. Sleep predicts morning affect and daily event ratings.

|  | **Predictors** | | | | | | | | | | | |
| --- | --- | --- | --- | --- | --- | --- | --- | --- | --- | --- | --- | --- |
| **Outcome Measures** | **Total Sleep Time** | | | | **Sleep Efficiency** | | | | **Sleep Quality** | | | |
| **Morning Affect** | *b* | *t* | *p* | *r* | *b* | *t* | *p* | *r* | *b* | *t* | *p* | *r* |
| Positive affect | .001 | 5.38 | < .001 | .18 | .004 | 1.71 | .088 | .12 | .20 | 8.69 | < .001 | .21 |
| Negative affect | -.001 | -5.57 | < .001 | .22 | -.006 | -3.11 | .002 | .17 | -.21 | -10.17 | < .001 | .21 |
|  |  | | | |  | | | |  |  |  |  |
|  | **Total Sleep Time** | | | | **Sleep Efficiency** | | | | **Sleep Quality** | | | |
| **Daily Events** | *b* | *t* | *p* | *r* | *b* | *t* | *p* | *r* | *b* | *t* | *p* | *r* |
| Peak Positivity of Positive Event | .003 | .95 | .345 | .02 | -.04 | -1.34 | .180 | .10 | .23 | .69 | .494 | .04 |
| Intensity of Positive Event (RA coded) | -.000 | -.35 | .726 | .05 | -.002 | -1.13 | .257 | .02 | .02 | .95 | .344 | .02 |
| Peak Stressfulness of Negative Event | -.02 | -3.58 | < .001 | .17 | -.037 | -.57 | .573 | .09 | -1.07 | -1.84 | .066 | .03 |
| Intensity of Negative Event (RA coded) | -.001 | -2.77 | .006 | .07 | -.002 | -1.14 | .253 | .04 | -.02 | -1.15 | .252 | .02 |

Supplemental Table 3. Evening affect and daily events predict sleep on that evening.

|  | **Predictors** | | | | | | | |
| --- | --- | --- | --- | --- | --- | --- | --- | --- |
|  | **Evening Positive Affect** | | | | **Evening Negative Affect** | | | |
| **Outcome Measures** | *b* | *t* | *p* | *r* | *b* | *t* | *p* | *r* |
| Total Sleep Time | 1.67 | .74 | .461 | .11 | -6.53 | -2.05 | .041 | .04 |
| Sleep Efficiency | .57 | 2.43 | .017 | .06 | -.37 | -.87 | .387 | .11 |
| Sleep Quality | .01 | .57 | .569 | .10 | -.13 | -3.39 | .001 | .13 |
|  |  |  |  |  |  |  |  |  |
|  | **Peak Positivity of Positive Event** | | | | **Intensity of Positive Event (RA coded)** | | | |
| **Outcome Measures** | *b* | *t* | *p* | *r* | *b* | *t* | *p* | *r* |
| Total Sleep Time | .08 | .75 | .456 | .10 | -1.02 | -.50 | .620 | .04 |
| Sleep Efficiency | .00 | .22 | .825 | .05 | -.05 | -.20 | .843 | .11 |
| Sleep Quality | .00 | .86 | .390 | .02 | .02 | 1.12 | .263 | .05 |
|  |  |  |  |  |  |  |  |  |
|  | **Peak Stressfulness of Negative Event** | | | | **Intensity of Negative Event (RA coded)** | | | |
| **Outcome Measures** | *b* | *t* | *p* | *r* | *b* | *t* | *p* | *r* |
| Total Sleep Time | -.06 | -.93 | .352 | .08 | 2.79 | 1.45 | .146 | .04 |
| Sleep Efficiency | -.00 | -.07 | .942 | .03 | .18 | .75 | .457 | .09 |
| Sleep Quality | -.001 | -1.81 | .070 | .11 | .01 | .25 | .800 | .12 |

Supplemental Table 4. Sleep predicts morning affect and daily event ratings.

|  | **Predictor** | | | | | |
| --- | --- | --- | --- | --- | --- | --- |
| **Outcome Measures** | **Total Sleep Time** | | | | | |
| **Morning Affect** | *b* [95% CI] | *se* | *p* | *r_w_*^(^*^f^*^)^ | *r_w_*^(^*^f1^*^)^ | *r_w_*^(^*^fv^*^)^ |
| Positive affect | .001 [.001, .002] | .000 | < .001 | .18 | .11 | .14 |
| Negative affect | -.001 [-.002, -.001] | .000 | < .001 | .22 | .12 | .18 |
|  | **Sleep Efficiency** | | | | | |
| Positive affect | .002 [-.003, .006] | .002 | .390 | .12 | .03 | .12 |
| Negative affect | -.007 [-.003, -.012] | .002 | < .001 | .17 | .08 | .15 |
|  | **Sleep Quality** | | | | | |
| Positive affect | .200 [.154, .245] | .023 | < .001 | .21 | .17 | .12 |
| Negative affect | -.223 [-.271, -.176] | .024 | < .001 | .21 | .18 | .11 |
|  |  |  |  |  |  |  |
|  | **Total Sleep Time** | | | | | |
| **Daily Events** | *b* [95% CI] | *se* | *p* | *r_w_*^(^*^f^*^)^ | *r_w_*^(^*^f1^*^)^ | *r_w_*^(^*^fv^*^)^ |
| Peak Positivity of Positive Event | .005 [-.003, .012] | .004 | .258 | .02 | .02 | .01 |
| Intensity of Positive Event (RA coded) | -.000 [-.000, .000] | .000 | .962 | .05 | .01 | .05 |
| Peak Stressfulness of Negative Event | -.023 [-.039, -.007] | .008 | .005 | .17 | .07 | .15 |
| Severity of Negative Event (RA coded) | -.001 [-.001, -.000] | .000 | .006 | .07 | .06 | .05 |
|  | **Sleep Efficiency** | | | | | |
| Peak Positivity of Positive Event | -.055 [-.132, .022] | .039 | .168 | .10 | .03 | .10 |
| Intensity of Positive Event (RA coded) | -.003 [-.001, .007] | .002 | .140 | .02 | .02 | .01 |
| Peak Stressfulness of Negative Event | -.015 [-.163, .133] | .076 | .841 | .09 | .01 | .08 |
| Severity of Negative Event (RA coded) | -.001 [-.005, .003] | .002 | .657 | .04 | .02 | .03 |
|  | **Sleep Quality** | | | | | |
| Peak Positivity of Positive Event | .005 [-.671, .680] | .345 | .990 | .04 | .01 | .04 |
| Intensity of Positive Event (RA coded) | .02 [-.022, .054] | .019 | .404 | .02 | .02 | .00 |
| Peak Stressfulness of Negative Event | -1.36 [-2.571, -.140] | .620 | .031 | .03 | .03 | .01 |
| Severity of Negative Event (RA coded) | -.02 [-.061, .014] | .019 | .227 | .02 | .02 | .01 |

Note: Effect sizes, *r_w_*^(^*^f^*^)^, were calculated following recommendations by Rights and Sterba (2019). The *r_w_*^(^*^f^*^)^ statistic is defined as the square root of the proportion of variance explained by within-person predictors via fixed slopes and random slope variation/covariation. This is similar to a measure of the square root of the proportion reduction in variance, akin to a correlation, but it may not correspond intuitively to *t*-values and *p*-values. The *r_w_*^(^*^f1^*^)^ statistic refers to the variance associated specifically with the fixed effects, whereas the *r_w_*^(^*^fv^*^)^ statistic refers to the variance associated with the random slope variation/covariation. The *r_w_*^(^*^f1^*^)^ statistic more closely aligns with the *t*-values and *p*-values, but we reported the *r_w_*^(^*^f^*^)^ statistic in the manuscript because that statistic most closely corresponds to the effect size estimates that have been used in prior research.

Supplemental Table 5. Evening affect and daily events predict sleep on that evening.

|  | **Predictor** | | | | | |
| --- | --- | --- | --- | --- | --- | --- |
|  | **Evening Positive Affect** | | | | | |
| **Outcome Measures** | *b* [95% CI] | *se* | *p* | *r_w_*^(^*^f^*^)^ | *r_w_*^(^*^f1^*^)^ | *r_w_*^(^*^fv^*^)^ |
| Total Sleep Time | .604 [-4.149, 5.356] | 2.425 | .804 | .11 | .02 | .11 |
| Sleep Efficiency | .308 [-.161, .776] | .239 | .199 | .06 | .05 | .04 |
| Sleep Quality | .021 [-.026, .068] | .024 | .388 | .10 | .01 | .10 |
|  |  |  |  |  |  |  |
|  | **Evening Negative Affect** | | | | | |
| Total Sleep Time | -1.842 [-8.847, 5.163] | 3.574 | .607 | .04 | .04 | .01 |
| Sleep Efficiency | -.243 [-1.061, .575] | .417 | .562 | .11 | .02 | .11 |
| Sleep Quality | -.143 [-.224, -.064] | .041 | .001 | .13 | .07 | .11 |
|  |  |  |  |  |  |  |
|  | **Peak Positivity of Positive Event** | | | | | |
| Total Sleep Time | .084 [-.130, .298] | .109 | .440 | .10 | .02 | .10 |
| Sleep Efficiency | .003 [-.020, .026] | .012 | .792 | .05 | .01 | .05 |
| Sleep Quality | .000 [-.002, .003] | .001 | .698 | .02 | .02 | .01 |
|  |  |  |  |  |  |  |
|  | **Intensity of Positive Event (RA coded)** | | | | | |
| Total Sleep Time | .643 [-3.686, 4.972] | 2.209 | .771 | .04 | .01 | .03 |
| Sleep Efficiency | .050 [-.404, .505] | .232 | .828 | .11 | .00 | .11 |
| Sleep Quality | .027 [-.016, .070] | .022 | .218 | .05 | .02 | .04 |
|  |  |  |  |  |  |  |
|  | **Peak Stressfulness of Negative Event** | | | | | |
| Total Sleep Time | .015 [-.110, .139] | .064 | .817 | .08 | .02 | .08 |
| Sleep Efficiency | .006 [-.007, .020] | .007 | .360 | .03 | .00 | .03 |
| Sleep Quality | -.001 [-.003, -.000] | .001 | .033 | .11 | .03 | .11 |
|  |  |  |  |  |  |  |
|  | **Severity of Negative Event (RA coded)** | | | | | |
| Total Sleep Time | 3.862 [-.222, 7.947] | 2.084 | .065 | .04 | .03 | .03 |
| Sleep Efficiency | .396 [-.097, .889] | .251 | .118 | .09 | .01 | .09 |
| Sleep Quality | .005 [-.039, .048] | .022 | .837 | .12 | .01 | .12 |

**Evening Affect Predicts Sleep**

In addition to the analyses presented in the main text, we considered the unique predictive effects of evening affect on sleep by entering each affective variable in the same model. The model is presented below. These results yielded similar conclusions to those reported in the main text.

Day level: *y_ijk_* (sleep) = *π*_0_*_jk_* + *π*_1_*_jk_* (evening positive affect) + *π*_2_*_jk_* (evening negative affect) + *e_ijk_*

Burst level*:* π_0_*_jk_* = β_00_*_k_* + *r*_0_*_jk_*

π_1_*_jk_* = β_10_*_k_* + *r*_1_*_jk_*

π_2_*_jk_* = β_20_*_k_* + *r*_2_*_jk_*

Person level: β_00k_ = γ_000_ + *u*_00_*_k_*

β_10k_ = γ_100_ + *u*_10_*_k_*

β_20k_ = γ_200_ + *u*_20_*_k_*

Supplemental Table 6. Positive and Negative Evening Affect Predicting Sleep.

|  | **Predictors** | | | | | | |
| --- | --- | --- | --- | --- | --- | --- | --- |
|  | **Evening Positive Affect** | | |  | **Evening Negative Affect** | | |
| **Outcome Measures** | *b* | *t* | *p* |  | *b* | *t* | *p* |
| Total Sleep Time | -.06 | -.02 | .981 |  | -2.55 | -.66 | .513 |
| Sleep Efficiency | .33 | 1.27 | .205 |  | -.03 | -.07 | .943 |
| Sleep Quality | -.01 | -.50 | .615 |  | -.15 | -3.32 | .001 |

Finally, we created separate evening affect measures by distinguishing activated and deactivated states. Positive activated affect was assessed with the following items: “I felt amused, fun-loving, silly,” “I felt excited, eager, enthusiastic,” “I felt inspired, uplifted, elevated,” “I felt interested, alert, curious,” and “I felt joyful, glad, happy.” Positive deactivated affect was assessed with the following items: “I felt awe, wonder, amazement,” “I felt in control, coping well, on top of things,” “I felt grateful, appreciative, thankful,” “I felt hopeful, optimistic, encouraged,” “I felt love, closeness, trust,” “I felt proud, confident, self-assured,” and “I felt satisfied, fulfilled, content.” Negative activated affect was assessed with the following items: “I felt angry, irritated, frustrated,” “I felt contemptuous, scornful, disdainful,” “I felt disgust, distaste, revulsion,” “I felt hatred, distrust, suspicion,” “I felt scared, fearful, afraid,” and “I felt stressed, anxious, overwhelmed.” Negative deactivated affect was assessed with the following items: “I felt ashamed, humiliated, disgraced,” “I felt bored, disinterested, uninvolved,” I felt embarrassed, self-conscious, blushing,” “I felt guilty, remorseful, blameworthy,” “I felt rejected, betrayed, left-behind,” and “I felt sad, downhearted, unhappy.” Reliabilities for positive activated affect, positive deactivated affect, negative activated affect, and negative deactivated were not as high as the composite scores (.78, .69, .47, and .58, respectively). We created models that included each affective measure in separate models. The results presented in Supplemental Table 7 show that activated and deactivated states had similar patterns of relationships with sleep outcomes.

Day level: *y_ijk_* (sleep) = *π*_0_*_jk_* + *π*_1_*_jk_* (evening affect) + *e_ijk_*

Burst level*:* π_0_*_jk_* = β_00_*_k_* + *r*_0_*_jk_*

π_1_*_jk_* = β_10_*_k_* + *r*_1_*_jk_*

Person level: β_00k_ = γ_000_ + *u*_00_*_k_*

β_10k_ = γ_100_ + *u*_10_*_k_*

Supplemental Table 7. Evening affect as a circumplex model predicting sleep.

|  | **Predictors** | | | | | | | | | | | | | | |
| --- | --- | --- | --- | --- | --- | --- | --- | --- | --- | --- | --- | --- | --- | --- | --- |
|  | **Evening Positive Activated Affect** | | |  | **Evening Positive Deactivated Affect** | | |  | **Evening Negative Activated Affect** | | |  | **Evening Negative Deactivated Affect** | | |
| **Outcome Measures** | *b* | *t* | *p* |  | *b* | *t* | *p* |  | *b* | *t* | *p* |  | *b* | *t* | *p* |
| Total Sleep Time | -.24 | -.12 | .904 |  | 1.38 | .54 | .588 |  | -4.38 | -1.50 | .135 |  | 1.50 | .41 | .685 |
| Sleep Efficiency | .25 | 1.23 | .221 |  | .30 | 1.23 | .220 |  | -.28 | -.81 | .419 |  | -.03 | -.08 | .933 |
| Sleep Quality | .01 | .73 | .469 |  | .02 | .83 | .410 |  | -.12 | -3.59 | < .001 |  | -.10 | -2.50 | .013 |
